# Predicting severity in COVID-19 disease using sepsis blood gene expression signatures

**DOI:** 10.1038/s41598-023-28259-y

**Published:** 2023-01-23

**Authors:** Arjun Baghela, Andy An, Peter Zhang, Erica Acton, Jeff Gauthier, Elsa Brunet-Ratnasingham, Travis Blimkie, Gabriela Cohen Freue, Daniel Kaufmann, Amy H. Y. Lee, Roger C. Levesque, Robert E. W. Hancock

**Affiliations:** 1 Department of Microbiology and Immunology, University of British Columbia (UBC), Vancouver, Canada; 2 Asep Medical, Vancouver, Canada; 3 Department of Molecular Biology and Biochemistry, Simon Fraser University, Burnaby, Canada; 4 Institut de Biologie Intégrative et des Systèmes (IBIS), Département de Microbiologie-Infectiologie et d’immunologie, Université Laval, Quebec, QC Canada; 5 Département de Microbiologie, Infectiologie Et Immunologie, Université de Montréal, Montreal, Canada; 6 Centre de Recherche du CHUM, Montreal, QC Canada; 7 Department of Statistics, UBC, Vancouver, Canada; 8 Département de Médecine, Université de Montréal, Montreal, Canada

**Keywords:** Predictive medicine, Computational biology and bioinformatics, Biomarkers, Infection, Inflammation

## Abstract

Severely-afflicted COVID-19 patients can exhibit disease manifestations representative of sepsis, including acute respiratory distress syndrome and multiple organ failure. We hypothesized that diagnostic tools used in managing all-cause sepsis, such as clinical criteria, biomarkers, and gene expression signatures, should extend to COVID-19 patients. Here we analyzed the whole blood transcriptome of 124 early (1–5 days post-hospital admission) and late (6–20 days post-admission) sampled patients with confirmed COVID-19 infections from hospitals in Quebec, Canada. Mechanisms associated with COVID-19 severity were identified between severity groups (ranging from mild disease to the requirement for mechanical ventilation and mortality), and established sepsis signatures were assessed for dysregulation. Specifically, gene expression signatures representing pathophysiological events, namely cellular reprogramming, organ dysfunction, and mortality, were significantly enriched and predictive of severity and lethality in COVID-19 patients. Mechanistic endotypes reflective of distinct sepsis aetiologies and therapeutic opportunities were also identified in subsets of patients, enabling prediction of potentially-effective repurposed drugs. The expression of sepsis gene expression signatures in severely-afflicted COVID-19 patients indicates that these patients should be classified as having severe sepsis. Accordingly, in severe COVID-19 patients, these signatures should be strongly considered for the mechanistic characterization, diagnosis, and guidance of treatment using repurposed drugs.

## Introduction

In the ongoing COVID-19 pandemic (2020–2022), it is increasingly evident that many of the millions of individuals who die with severe disease succumb due to sepsis, a life-threatening dysfunctional response to infection. Studies show that many or most individuals who died from severe COVID-19 infections had acute respiratory distress syndrome (ARDS) and high Sequential Organ Failure Assessment (SOFA) scores (ranging from 2.6 to 12 among patients with life-threatening disease)^1,2^. Furthermore, more extensive phenotyping studies have suggested that features of sepsis such as considerable organ damage (e.g., lung and kidney injury) and long-term depressed mental health and immune responses are prevalent in severe COVID-19 patients^3,4^. The possibility has been discussed that this is viral-driven sepsis^5^, but the involvement of bacterial infections in at least 50% of severely-afflicted COVID-19 patients has also been established^6,7^.

The characterization of COVID-19 disease as a type of sepsis has important clinical implications for the prognosis and treatment of severely-afflicted COVID-19 patients^8^. The clinical criteria (e.g., SOFA, Multiple Organ Dysfunction Score [MODS]), biomarkers, and immunomodulatory therapies proposed for the management of sepsis can likely be generalized to COVID-19 if the severe manifestation of this disease is a type of sepsis. Unfortunately, sepsis itself has proven difficult to define using clinical criteria, since symptomatology ranges from non-specific/mild presentation to organ dysfunction, the requirement of mechanical ventilation and life support, and death^9^. Whole blood transcriptomics has proven useful in characterizing dysregulated host immune responses during sepsis and, importantly, has provided novel opportunities to define the sepsis syndrome using multi-molecule signatures^10^. These signatures have enabled the development of more specific criteria to identify, diagnose, and categorize sepsis patients. For example, we recently characterized, in emergency room (ER) patients, a set of gene expression signatures reflective of sepsis endotypes (a recent paradigm to separate patients into coherent and mechanistically distinct subgroups) as well as endotype-independent global markers of severity predictive of impending organ dysfunction and mortality^11,12^.

Many studies have used transcriptomics to characterize the specific gene expression events dysregulated in severe COVID-19 patients, like those performed in all-cause sepsis contexts. These have suggested that COVID-19 specific signatures/mechanisms, in part reflecting innate immune responses and canonical hallmarks of inflammation in neutrophils, monocytes, and T cells, can likely be exploited for diagnostic and prognostic value in COVID-19 disease^13^. However, far fewer studies have explored the gene expression profiles of severe COVID-19 patients in the context of known molecules dysregulated in all-cause (but frequently bacterial-infection triggered) sepsis. This would provide an explicit link to the underlying biological programs defining the sepsis syndrome. In this study, we recruited patients with confirmed COVID-19 infection and a broad range of disease severity from various centers in Quebec, Canada to determine if COVID-19 patients displayed a significant dysregulation of established signatures. We show that all-cause sepsis signatures, specifically those reflecting cellular reprogramming (CR), organ dysfunction, and mortality, are mediators of COVID-19 pathogenesis and discriminate between COVID-19 severity groups. Furthermore, COVID-19 patients fit into the same mechanistic groupings/endotypes that are evident in all-cause sepsis. These results suggest that mechanisms underlying the severity of COVID-19 are those found in sepsis, and can be represented by the specific gene expression signatures that define this syndrome.

## Methods

### Study design and patient recruitment

We sought to characterize global transcriptional changes that occur through a range of severities from moderate to severe COVID-19 disease (as defined by requirement for respiratory support) and to determine whether these changes overlapped with those observed during all-cause sepsis. The Biobanque Québécoise de la COVID-19 (BQC-19; Quebec COVID-19 Biobank; https://en.quebeccovidbiobank.ca/) recruited, in 10 hospitals, adult and pediatric patients (across ERs, wards, ICU, and outpatient clinics) with confirmed SARS-CoV-2 infection in a province-wide initiative to collect, store, and share samples and data related to the Covid-19 pandemic^14^. The study was approved by the respective institutional review boards and written informed consent was obtained from all participants or, when incapacitated, their legal guardian before enrollment and sample collection.

COVID-19 severity was assessed by the extent of respiratory support received at sampling, with patients assigned to groups comprising severe (evidence of organ failure and required ECMO, mechanical ventilation, or non-invasive ventilation), intermediate (needed supplemental oxygen through a nasal cannula), or moderate (required no supplemental oxygen and had milder symptoms), as well as recording eventual mortality. To enable retrospective association with gene expression data, various clinical and demographic metadata were collected, including age, sex, duration of hospital stay, non-COVID active infections, etc. (Table 1). The Clinical Research Ethics Board of the University of British Columbia (UBC; approval number H17-01208) and Comité d’éthique de la recherche du Centre hospitalier de l’Université de Montréal (CUM-CHUM; approval numbers MP-02-2020-8929 and 19.389) provided ethics approval for all sequencing and bioinformatics studies, carried out in a manner blinded to patient identity. All methods described were performed in accordance with the relevant guidelines and regulations.

**Table 1:** Sepsis severity and outcomes of the cohorts with confirmed COVID-19.

Parameter	Quebec hospital-wide (wards and ICU) cohort (N = 124)
Age	61.3 ± 1.35 (124)
Sex, female	38.7% (48/124)
Duration of illness before hospital arrival (days)	6.6 ± 0.37 (118)
Hospital stay duration (days)	23.2 ± 2.0 (124)
On Antibiotics	80.0% (96/120)
Non-COVID active infection	35.8% (44/123)
Sampling group	Early 61.3% (76 patients); late 38.7% (48 patients)
Existing lung disease	10.5% (13/124)
Existing kidney disease	20.2% (25/124)
Existing heart disease	11.2% (13/124)
Existing liver disease	0.81% (1/124)
Respiratory support at sampling	Severe, 43.5% (54/124); intermediate, 22.6% (28/124); moderate, 33.9% (42/124)
SOFA scores	8.02 ± 0.54 (54)
ICU admission	44.4% (55/124)
Mortality (in hospital)	16.1% (20/124)

The mean value ± standard error is presented for numerical variables (total observations in brackets). Categorical variables are presented as % positive observations (relative to total observations). Early samples were taken 1–5 days post admission while late samples 6–20 days post admission. Numbers in brackets are the numbers of patients in each category. Respiratory support definition—severe: required extracorporeal membrane oxygenation (ECMO), Intubation, or Non-invasive ventilation; intermediate: required supplemental oxygen through nasal cannula; moderate: no supplemental oxygen required.

### Blood collection and transcriptome sequencing (RNA-Seq)

During the usual blood sample collections in hospital patients, an additional 2.5 ml of blood was collected for RNA-Seq. Blood was collected into PAXgene Blood RNA tubes (BD Biosciences; San Jose, CA, USA) to ensure stabilization of intracellular RNA^15^. Total RNA was extracted using the PAXgene Blood RNA Kit (Qiagen; Germantown, MD, USA). Quantification and quality measures for total RNA were obtained using a LabChip GXII instrument (Waltham, MA, USA). Poly-adenylated RNA was captured using NEBNext Poly(A) mRNA Magnetic Isolation Module (NEB; Ipswich, USA) and cDNA libraries were prepared using the NEBNext RNA First Strand Synthesis Module, NEBNext Ultra Directional RNA Second Strand Synthesis Module, and NEBNext Ultra II DNA Library Prep Kit for Illumina (NEB; Ipswich, USA). RNA-Seq was then performed at a depth of 50 M reads/sample on an Illumina NovaSeq 6000 S4 instrument of 100 base-pair long paired-end sequence reads (excluding adapter/index sequences).

A standard data processing protocol was used, including quality control using fastqc (v0.11.9)^16^ and multiqc (v1.6)^17^, alignment to the human genome (Ensembl GRCh38.104) using STAR (v2.7.9a)^18^, and read count assessments using htseq-count (v0.11.3)^19^. Finally, globin genes and genes with fewer than 10 counts were removed from count tables, and we ensured that analyzed samples had more than one million total read counts. Raw sequencing data are available at NCBI GEO (Accession Number GSE221234). When performing an analysis other than differential gene expression analysis (which typically requires raw counts as input), the variance stabilizing transformation (VST) from the DESeq2 (v1.22.2) R package was performed to render counts homoscedastic and normalized for varying library sizes^20^. Following transformation, technical variation due to sequencing batch was removed using ComBat within the Surrogate Variable Analysis R package (v3.30.1)^21^.

### Bioinformatics and statistical analysis

Mechanisms of COVID-19 severity were determined using differential expression analysis by comparing severity groups based on the respiratory support required and impending mortality. Differential expression analysis was performed using DESeq2, with genes considered differentially expressed (DE) if they displayed ≥  ± 1.5-fold change and Benjamini–Hochberg adjusted p value ≤ 0.05 (Wald test). Functional characterization of DE genes was performed using an over-representation/enrichment analysis of Reactome pathways^22^ and MSigDB hallmarks^23^. A pathway/hallmark was considered enriched if it displayed an adjusted p value ≤ 0.05 (hypergeometric test).

We explored whether these patients demonstrated dysregulation of previously-described all-cause sepsis severity signatures^12,24^. These signatures were identified in a geographically diverse cohort of early all-cause sepsis patients and further validated in two independent cohorts of ER and ICU patients (including non-Covid-19/bacterial and Covid-19 sepsis patients). This allowed us to validate the use of all-cause sepsis signatures and further examine whether severe COVID-19 is a type of sepsis. The supervised DE gene-set algorithm Rotation Gene Set Test (ROAST), provided within the limma R package (v3.38.3)^25,26^, was used to assess whether these signatures were significantly dysregulated between COVID-19 severity groups.

Sepsis endotypes were also examined within the data^12^. Endotypes are subtypes of a disease defined by unique pathophysiology and underlying mechanisms. Predicting endotype status in the current cohort involved calculating a sample-wise gene-set enrichment score for each independent 200-gene endotype signature. Specifically, the over- or under-expression of signatures in individual patients was estimated using the GSVA R package (v1.30.0); this assessed expression differences of a gene set when compared to all other genes (i.e., genes not in the set) within each sample/patient^27^. Then patients were classified to the endotype exhibiting the highest expression or enrichment of the respective endotype signature. Clinical parameters were statistically compared between predicted endotypes using Kruskal–Wallis and Chi-Squared tests to compare numerical and categorical variables, respectively.

The funders (Canadian Institutes for Health Research; FDN-154287) and the Fonds de recherche du Québec (FRQ) COVID-19 Biobank had no role in the manuscript's analysis, interpretation, or writing.

## Results

### Mechanisms of sepsis severity and mortality in COVID-19 patients

To identify COVID-19 specific severity mechanisms, we initially compared the whole blood gene expression profiles associated with defined severity groups from a cohort of 124 patients recruited at various times relative to hospital admission. Patient severity was assessed using two measures, one based on the requirement for respiratory support and the second based on whether a patient died in hospital. Based on principal component analysis (PCA) of global gene expression profiles, patients could be well separated according to respiratory support requirement group and eventual in-hospital mortality on two dimensions (Supplemental Fig. 1a). The association of several parameters (including respiratory support requirement group, various comorbidities, and time of sampling relative to hospital admission) with gene expression principal components was also explored using multiple linear regression (Supplemental Fig. 1b). These results indicated strong association with the second principal component, PC2, for eventual mortality (p value = 0.000059; multiple linear regression) and respiratory support group (p value = 0.0062), with PC2 explaining 17.8% of the total gene expression variance. Interestingly, other influences on gene expression, including sex, non-COVID-19 active infections, active cancer diagnosis, etc., were not associated with the most variable PCs (i.e., PC1-4).

**Figure 1:**
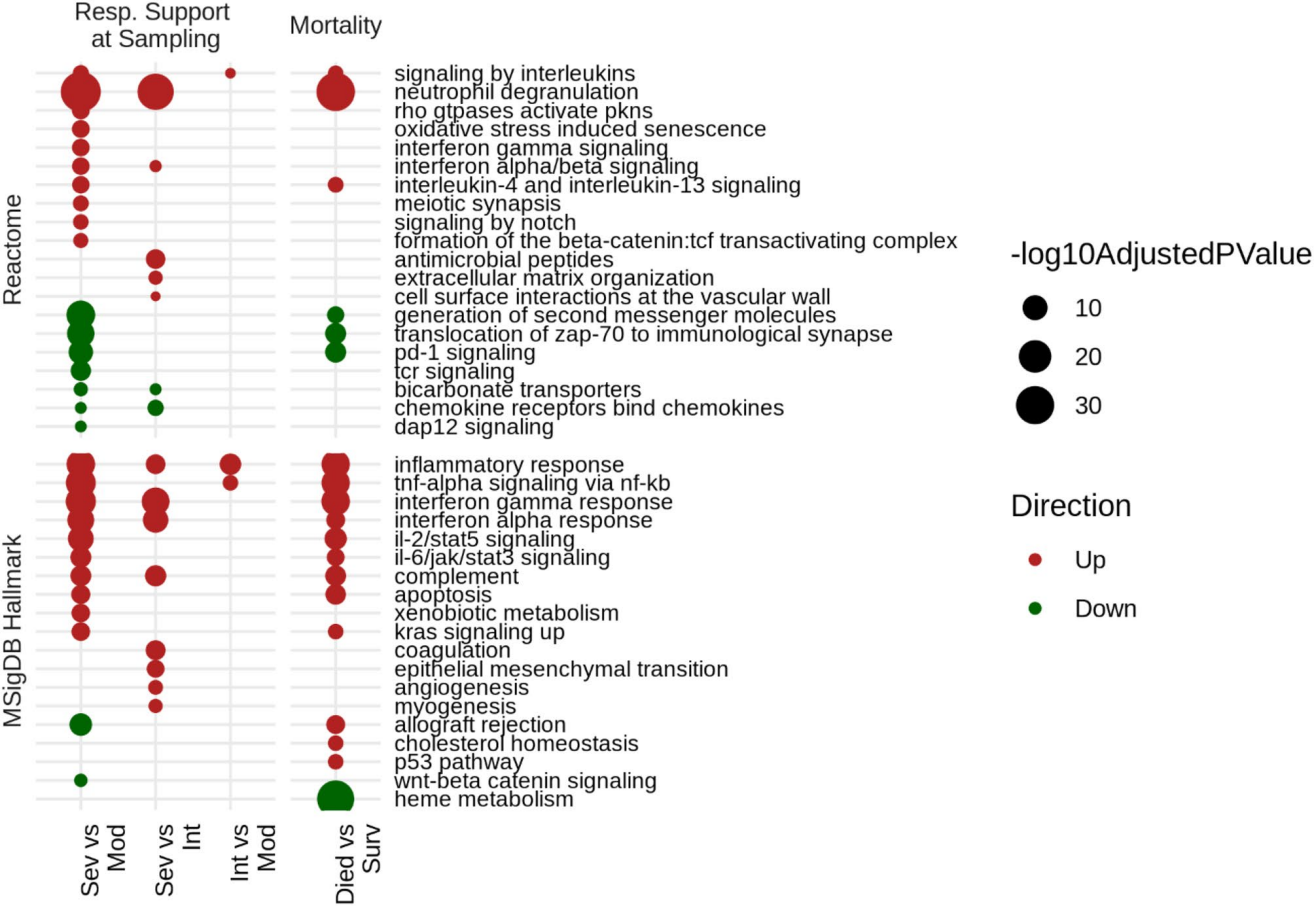
Functional characterization of the gene expression differences between severity groups and eventual mortality in Quebec COVID-19 patients. Shown are the top 10 enriched pathways/hallmarks for each comparison, direction, and gene set database assessed (i.e., lowest p values). Severity groups were assessed using the respiratory support required at time of sampling with 54 severe, 28 intermediate, and 42 moderate patients. There were 20 patients who died and 100 patients who survived. The severe vs. moderate, severe vs. intermediate, and intermediate vs. moderate, died vs. survived, comparisons yielded 2671 (1794 up, 877 down), 694 (535 up, 159 down), 169 (136 up, 33 down), and 1708 (1006 up, 702 down) DE genes, respectively (displaying ≥  ± 1.5-fold change; adjusted p ≤ 0.05). Age, sex, batch, and time of sampling relative to hospital admission were included in the underlying DESeq2 model to correct for their contribution to gene expression. A post-hoc power analysis (performed with the online tool RnaSeqSampleSize^53^) using parameters estimated from previous RNA-Seq studies, indicated our study was sufficiently powered (p = 0.8) to detect DE gene differences in gene expression (at least 15% of transcriptome) between patient groups.

Differential expression analysis comparing patient severity groups, as defined by need for respiratory support, indicated several thousands of genes were dysregulated in severe COVID-19 disease. Most notably, when comparing severe vs. moderate patients, there were 2671 differentially expressed (DE) genes, with 1794 up-regulated and 877 down-regulated (≥ ± 1.5-fold change; adjusted p ≤ 0.05). These were analyzed for over-representation of Reactome^22^ and Hallmark gene-sets^23^, which capture pathways and well-defined biological processes, respectively. Up-regulated genes differentiating severe vs. moderately ill patients were enriched in several pathways, including those involving neutrophil degranulation, interferon α/β/γ signaling, interleukin (IL)-4 and IL-13 signaling, oxidative stress, and signaling by NOTCH. Furthermore, canonical inflammation hallmarks such as TNF-α signaling via NFκ-B and the Inflammatory response were also enriched (Fig. 1). Down-regulated DE genes were enriched in other pathways/hallmarks, including adaptive immune pathways such as programmed cell death-1 (PD-1) signaling, generation of second messenger molecules, TCR signaling, and allograft rejection. These results indicated that while anti-viral responses were enhanced, T-cell responses and activity were likely reduced during severe COVID-19 disease, consistent with severe all-cause sepsis^12,28^. The severe vs. intermediate comparison also showed a significant dysregulation of gene expression (535 up-regulated, 159 down-regulated); with enriched pathways/hallmarks including neutrophil degranulation, interferon α/β/γ signalling, coagulation, and complement. The intermediate vs. moderate comparison yielded relatively few DE genes (136 up-regulated and 33 down-regulated) and enriched pathways/hallmarks, indicating these groups were most similar based on gene expression (or conversely that the intermediate group showed some heterogeneity).

When comparing patients who succumbed in the hospital to those who survived, there were 1006 up-regulated and 702 down-regulated genes. The majority of these genes overlapped with the DE genes obtained when comparing severe vs. moderate patients (1122 overlapping genes) (Fig. 1). As such, neutrophil degranulation, interleukin signaling, down-regulation of T cell signaling pathways, and inflammatory hallmarks were commonly enriched between respiratory support severity and eventual mortality comparisons. Interestingly, heme metabolism, cholesterol homeostasis, and p53 pathways were uniquely enriched in DE genes from the mortality comparison, indicating that additional mechanisms became prominently dysregulated when mortality was imminent. Indeed, heme metabolism was the most significantly enriched hallmark among genes DE between patients who died vs. survived, and was downregulated (Fig. 1). This suggests that the down-regulation of heme metabolism is likely a specific feature of severe Covid-19 disease and was most evident among patients who succumbed to this disease. We further examined gene expression data of previously published COVID-19 positive and negative patients from Toronto, Ontario, who were recruited on the first day of ICU admission for COVID-19 specific pathways (GEO accession GSE185263)^12^. Highly enriched interferon-α/β/γ signaling (up-regulated) and heme metabolism (down-regulated) was apparent in COVID-19 positive patients when compared to negative patients as well as in COVID-19 positive compared to uninfected healthy controls (Supplemental Fig. 2).

The common enrichment of the neutrophil degranulation pathway across severity comparisons indicated that neutrophil activity and/or abundance was a marked feature of severe COVID-19 sepsis. This feature has been described in previous studies of all-cause sepsis, with several neutrophil and granulocyte genes and pathways being dysregulated in all-cause sepsis patients progressing to organ dysfunction/failure^12,29^. The ratio of neutrophils to lymphocytes cellular abundance in patients has been touted as a moderately accurate (AUC ≈ 0.695) and easily measurable prognostic biomarker of sepsis severity and outcomes in patients with advanced sepsis^30^. We estimated the neutrophil to lymphocyte ratio using the gene expression-based deconvolution program CIBERSORT^31^ and from cell differential values measured in hospital in order to explore their association to respiratory support group (Supplemental Fig. 3). It was clear that patients with severe and intermediate COVID-19 had the highest neutrophil to lymphocyte ratio which significantly differed from that of moderate patients (p = 9.2 × 10^–7^ by the Kruskal–Wallis test using CIBERSORT estimated ratios).

Although our initial motivation was to identify COVID-19 specific markers of patient severity, it was apparent from our analyses that existing comorbidities in patients contributed to the observed gene expression values (Supplemental Fig. 1b), including chronic renal, lung and heart insufficiencies and active cancer. It is well-accepted that existing comorbidities influence the risk of sepsis and sepsis severity^32^, however, few studies have examined the role of comorbidities on the transcriptomic profiles in COVID-19 sepsis. Accordingly, we performed DE analysis, followed by pathway/hallmark analysis, to identify mechanisms dysregulated between COVID-19 patients with existing chronic insufficiencies vs. those without (Supplemental Fig. 4). The largest dysregulation observed was among patients with chronic lung insufficiencies, which is notable considering SARS-CoV-2 infection is characterized by progression of the infection into the lower respiratory tract (i.e., lung tissue). This comparison yielded down-regulated MSigDB hallmarks, including complement, coagulation, and inflammatory responses. Interestingly, patients with chronic renal insufficiencies, cf. those with no chronic renal issues, also exhibited dysregulation of unique mechanistic subsets, including an up-regulation of interferon α/β/γ signaling, which was also observed among comparisons of Covid-19 severity. When comparing those with chronic heart insufficiencies vs. not, few DE genes and pathways/hallmarks arose. These results indicate that overall specific gene expression differences occurred due to pre-existing comorbidities during COVID-19 disease, highlighting the possibility of adjusting therapeutic interventions according to comorbidity status.

### COVID-19 patients displayed dysregulation of all-cause sepsis severity signatures

Several mechanisms were dysregulated between COVID-19 severity groups, and many of these could be biomarkers of COVID-19 pathogenesis and thus valuable for patient prognostication. Some of these mechanisms have been implicated in sepsis, including those reflecting neutrophil activity, inflammatory cytokines, and adaptive immune pathways strengthening the notion that severe COVID-19 is a type of sepsis. To further indicate the extent to which severe COVID-19 overlaps with all-cause sepsis, we took the reverse approach, specifically determining whether prognostic gene expression signatures uncovered in all-cause sepsis cohorts are dysregulated in COVID-19 patients.

We previously described reduced 8–12 gene mechanistic signatures of sepsis severity uncovered in all-cause sepsis training cohorts^11,12^. These included the cellular reprogramming (CR; 8 genes), Organ Dysfunction (12 genes), and Mortality (10 genes) signatures, which were identified and validated in early sepsis patients at first clinical presentation to the ER as well as in the ICU. The CR and Organ Dysfunction signatures were shown to be highly accurate in predicting impending severe sepsis and organ dysfunction. Similarly, the Mortality signature was strongly associated with 30-day mortality and reasonably predictive of impending mortality. We determined whether these signatures were dysregulated between COVID-19 severity groups using ROAST, a supervised gene-set DE algorithm that assesses whether a set of genes, as a unit, is significantly up- or down-regulated^25^.

When analyzing all patients among the Quebec cohort, the CR, Organ Dysfunction, and Mortality signatures were strongly associated with the assessed endpoints (Table 2). In particular, when comparing severe vs. moderate patients each signature displayed p values < 0.0001, indicating a strong degree of signature dysregulation between these patient groups. This suggested that all-cause sepsis signatures were also highly relevant to COVID-19 pathogenesis in severely-afflicted patients. It is important to note that this cohort was comprised of patients sampled early and late in their hospital stays, and, as indicated in Supplemental Fig. 1b, sampling to admission time played a significant role in gene expression variability. Accordingly, we assessed the gene-set level DE of the severity signatures among patients’ groups separated by sampling time (i.e., early and late). This was done to minimize the role that disease progression might have had in the performance of the signatures. Patients were stratified into early (sampled within 1–5 days) and late (sampled between 6 and 20 days) post-hospital admission groups to assess whether gene expression signatures exhibited altered behaviour when accounting for this source of heterogeneity. The results indicated that across early and late sampled patients, the signatures were still significantly dysregulated between severe vs. moderate and dead vs. surviving patients. Severe vs. intermediate and intermediate vs. moderate patients exhibited lesser dysregulation, and this was most evident in late sampled patients. The gene set level dysregulation of the full CR, Organ Dysfunction, and Mortality signatures^12^ was also assessed (Supplemental Table 1), and displayed similar results to those obtained using the reduced signatures (Table 2).

**Table 2:** Gene-set differential expression of the reduced cellular reprogramming/CR (8 genes), organ dysfunction (12 genes), and mortality (10 genes) signatures in COVID-19 patient cohorts.

Cohort	Comparison	Statistical significance (p value)		
		CR	Organ dysfunction	Mortality
All	Severe (54) vs. moderate (42)	** < 0.0001**	** < 0.0001**	** < 0.0001**
	Severe (54) vs. intermediate (28)	** < 0.0001**	**0.0058**	0.063
	Intermediate (28) vs. moderate (42)	** < 0.0001**	**0.0021**	**0.0045**
	Died (20) vs. survived (104)	**0.0014**	**0.0023**	** < 0.0001**
Early sampled (0–5 days)	Severe (28) vs. moderate (29)	** < 0.0001**	**0.00040**	** < 0.0001**
	Severe (28) vs. intermediate (19)	0.053	0.47	**0.037**
	Intermediate (19) vs. moderate (29)	**0.00020**	**0.0057**	**0.025**
	Died (11) vs. survived (65)	**0.012**	**0.0095**	**0.00030**
Late sampled (6–20 days)	Severe (26) vs. moderate (13)	** < 0.0001**	** < 0.0001**	**0.0014**
	Severe (26) vs. intermediate (9)	**0.0090**	**0.0020**	0.44
	Intermediate (9) vs. moderate (13)	0.29	0.25	0.076
	Died (9) vs. survived (39)	**0.043**	0.13	**0.0043**

The gene sets were assessed for significant dysregulation between patients using ROAST. Statistically significant gene-set DE is indicated by bold typeface (FDR adjusted p ≤ 0.05).

**Figure 2:**
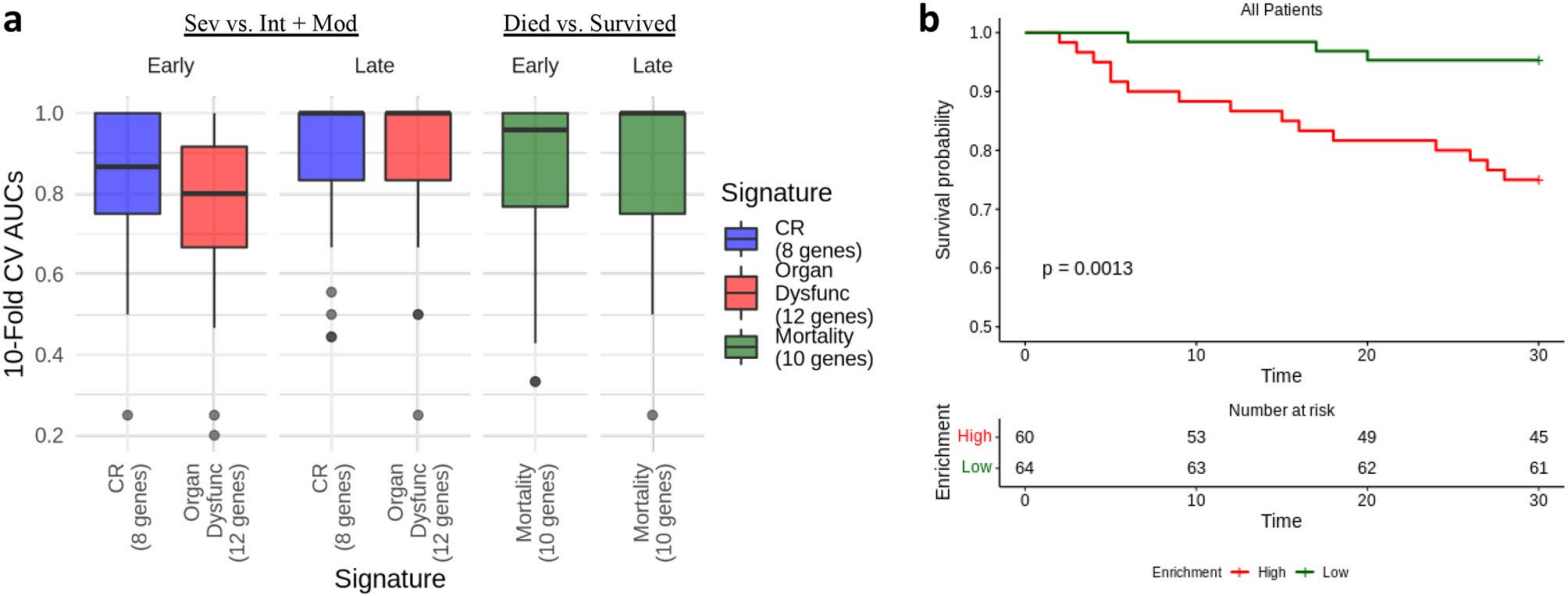
Predictive performance of the reduced CR, Organ Dysfunction, and Mortality signatures among patients of the COVID-19 patient cohorts. (**a**) Accuracy/AUCs across a tenfold repeated cross validation scheme (i.e., total 100 folds) using logistic regression. (**b**) Kaplan Meier curves of patients with High or Low Mortality signature enrichment in all patients (i.e., early and late sampled patients). Time to mortality was assessed over 30 days, with the baseline time being the day of sampling. Patients were stratified into either High (score ≥ 0; n = 60) or Low (score < 0; n = 64) signature expression based on GSVA enrichment statistics, and this was used as a predictor of 30-day survival.

We also determined the predictive accuracy of the CR, Organ Dysfunction, and Mortality signatures when used to directly predict patient severity groups. Logistic regression models, for which the reduced signature gene sets provided input into the model as covariates/predictors (Fig. 2a), were trained to predict respiratory support group and eventual mortality. In the case of respiratory support group, a binary classification scheme was trained to predict severe vs. intermediate + moderate patients. We combined the intermediate and moderate patients because their gene expression profiles were most similar when analyzed using differential expression analysis. Due to the modest size of these predictive reduced signatures (8–12 genes), they reflected subsets with the greatest potential for translation, since smaller signatures can be more easily assessed in clinics using qRT-PCR. A tenfold repeated cross validation was used to estimate predictive performance. The Organ Dysfunction (mean AUC = 0.79, sensitivity = 0.71, specificity = 0.68) and CR (mean AUC = 0.86, sensitivity = 0.80, specificity = 0.79) signatures displayed good performance in early sampled patients. Among late sampled patients, the Organ Dysfunction (mean AUC = 0.92, sensitivity = 0.80, specificity = 0.77) and CR (mean AUC = 0.91, sensitivity = 0.83, specificity = 0.81) signatures also displayed excellent performance.

The Mortality signature also displayed good performance in predicting eventual mortality in early (mean AUC = 0.86, sensitivity = 0.77, specificity = 0.75) and late (mean AUC = 0.89, sensitivity = 0.78, specificity = 0.86) sampled patients. We also explored the association of the Mortality signature with 30-day mortality using Kaplan Meier survival curves across all patients. The patient-level enrichment/expression scores were estimated for the Mortality signatures using GSVA that summarizes the expression of a gene set when compared to all other genes (i.e., genes not in the set) within each sample. Patients were separated into High (enrichment score ≥ 0; n = 60) and Low (enrichment score < 0; n = 64) signature enrichment categories to test their association with 30-day mortality. There was a significant association of high mortality signature enrichment with 30-day mortality (full mortality signature: log-rank p value = 0.00062; reduced mortality signature: log-rank p value = 0.0013) (Fig. 2b).

### COVID-19 patients exhibited evidence of all-cause sepsis endotypes

Our group also recently described, in ER patients at first clinical presentation, five sepsis endotypes. Endotypes are distinct disease groupings characterized by unique pathobiological mechanisms and DE gene expression signatures^12^. Importantly, endotypes are hypothesized to accurately explain the heterogeneous nature of the sepsis syndrome in contrast to earlier paradigms considering sepsis as a single disease. The early sepsis endotypes included two poor prognosis endotypes, namely the Neutrophilic-Suppressive (NPS) and Inflammatory (INF) endotypes, that were respectively associated with neutrophil activation/immune suppression and increased pro-inflammatory responses. Three additional fair-prognosis endotypes were also uncovered, namely the Innate Host Defences (IHD), Interferon (IFN), and Adaptive (ADA) endotypes. To predict endotype status in other cohorts, five 200-gene endotype-specific signatures were derived. Evidence of sepsis endotypes among COVID-19 patients would further support the notion that severe COVID-19 disease is the same as, or similar to, all-cause sepsis.

**Figure 3:**
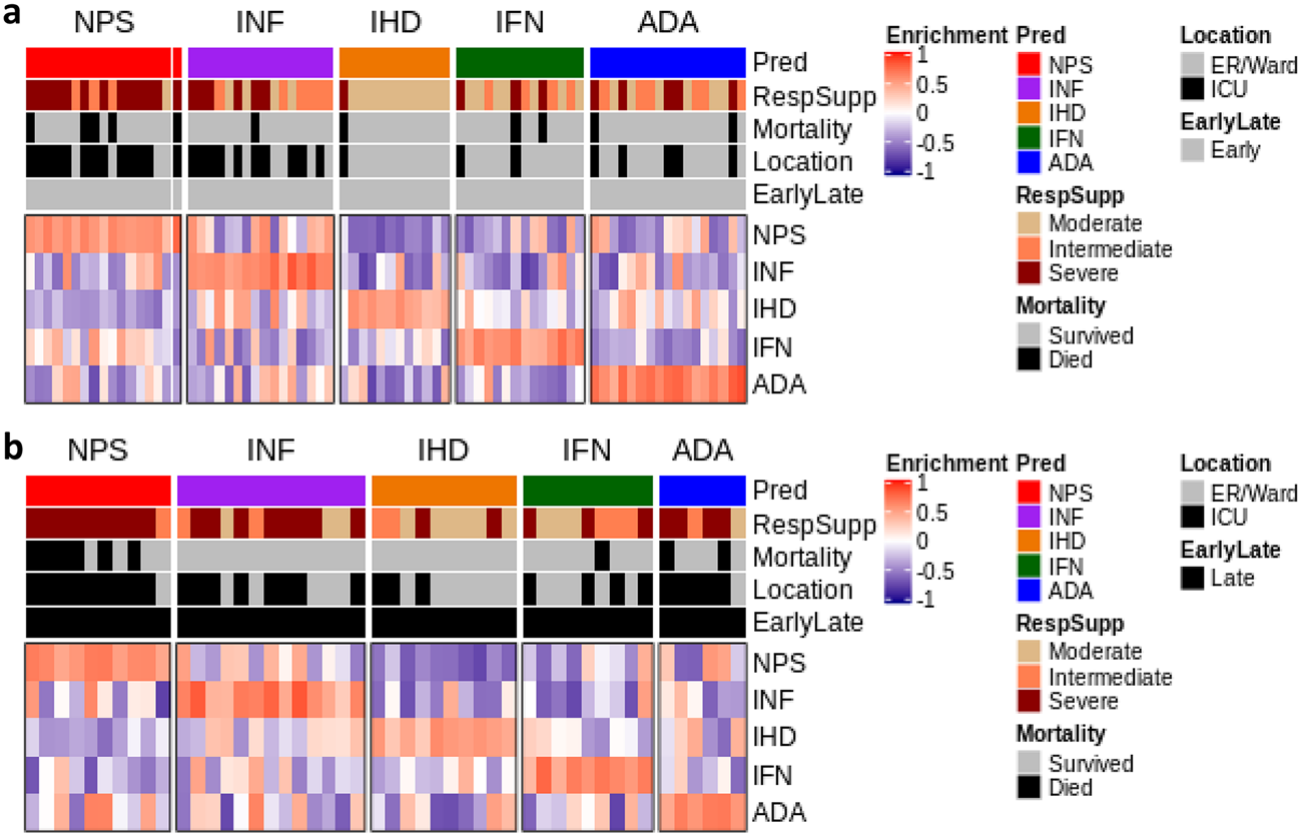
Heatmap depicting GSVA endotype signature enrichment scores and endotype classification COVID-19 patients. (**a**) Endotype classification of early sampled patients. (**b**) Endotype classification of late sampled patients. Endotype enrichment scores are indicated in the bottom five rows in each subfigure with red blocks indicating positive enrichment. Various metrics are indicated in the top five bars in each subgraph including endotype assignment, patient severity (as assessed by respiratory support requirement at sampling), impending mortality, and ICU admission, as well as Early (top subfigure) or Late (bottom subfigure) sampling. In both the early and late groups, the NPS endotype displayed the worst patient severity and highest mortality rates.

Initially, to characterize endotypes in patient whole blood RNA-Seq data, we determined, using a data-driven approach, whether the gene expression data supported the partitioning of these patients into 5 discrete clusters. Cluster stability metrics inferred that 4–6 clusters could be reliably discerned among all patients analyzed together and also among patients separated into early and late sampled groups (Supplemental Fig. 5). We next sought to classify patients into one of the five previously-described all-cause sepsis endotypes. A better assessment of severity signatures was obtained by stratifying patients into the early and late cohort patients, which partially resolved gene expression heterogeneity due to sampling time relative to hospital admission. Accordingly, endotype status was separately predicted in early and late sampled patients (Fig. 3) by calculating a sample-wise gene-set enrichment score for each 200-gene endotype-specific signature using GSVA (v1.30.0). Patients were then assigned to the endotype for which they exhibited the highest expression/enrichment score; this is depicted in Fig. 3 as blocks of red (demonstrating enrichment) in the bottom five rows of each subfigure.

Across both early and late sampled patients, endotype classification worked well to separate patients into five endotypes of similar size. In early sampled patients, these (predicted) endotypes positively correlated with assignment to severe respiratory support requirement group (p value = 0.0015), qSOFA (p value = 0.045), and Glasgow Coma Score (p value = 0.017) (Table 3; Supplemental Table 2). The predicted NPS patients were primarily comprised of patients belonging to the severe respiratory support group (77%; 13/17) and also had the highest mortality, with 29.4% (5/17) of patients succumbing. The INF endotype also displayed a high number of patients belonging to the severe respiratory support group (37.5%; 6/16) in addition to the highest qSOFA scores (recorded early in hospitalization). In early sampled patients, the clinical trends of the predicted NPS and INF endotypes were generally consistent with the all-cause sepsis discovery cohort^12^. However, a subgroup of more severely afflicted patients (higher severity and mortality) was also evident in the IFN and ADA endotypes, which differed from trends observed in the smaller all-cause sepsis discovery and validation cohorts examined previously^12^. For example, the patients predicted as part of the generally less severe ADA endotype, displayed high proportions of severe respiratory support patients (29.4%; 5/17).

**Table 3:** Clinical differences between predicted endotypes in the early sampled group.

Parameter	NPS (17/76)	INF (16/76)	IHD (12/76)	IFN (14/76)	ADA (17/76)	p value
Respiratory support group—moderate	5.9% (1/17)	25% (4/16)	91.7% (11/12)	50.0% (7/14)	35.3% (6/17)	0.000075
Receiving ICU care	76.5% (13/17)	62.5% (10/16)	8.3% (1/12)	14.3% (2/14)	29.4% (5/17)	0.00018
Respiratory support group—severe	76.5% (13/17)	37.5% (6/16)	8.3% (1/12)	21.4% (3/14)	29.4% (5/17)	0.0015
Glasgow coma score	13.3 ± 1.71 (7)	8 ± 2.86 (5)	15 ± 0 (5)	15 ± 0 (5)	15 ± 0 (9)	0.0174
P/F ratio	213.6 ± 19.82 (7)	175.2 ± 29.91 (5)	382.6 ± 21.36 (5)	274.4 ± 58.3 (5)	254.8 ± 31.26 (9)	0.022
qSOFA	0.6 ± 0.3 (7)	1.4 ± 0.4 (5)	0 ± 0 (5)	0.2 ± 0.2 (5)	0.4 ± 0.18 (8)	0.0454

The mean value ± standard error is presented for numerical variables with the total available observations/patient numbers recorded in brackets. Categorical variables are presented as % positive (relative to total available observations). Significance was assessed using a Kruskal–Wallis and Chi Square Test for numeric and categorical parameters, respectively. The P/F ratio is the arterial pO_2_ (“P”) divided by the FIO_2_ (“F”) with a P/F Ratio less than 300 indicating acute respiratory failure^52^. A full table of clinical parameters assessed, including non-significant parameters, is provided in Supplemental Table 2**.**

In late sampled patients, the predicted NPS endotype patients again displayed the highest rates of mortality (60%) and ICU care (90%) (Supplemental Table 3). Beyond this observation, the patients assigned to other predicted endotypes showed less consistent clinical parameters relative to endotype assignment when compared to the early sampled patients (Table 3) and all-cause sepsis patients^12^, possibly reflecting the fact that these endotypes were originally predicted at first clinical presentation (ER prior to ICU admission and any diagnosis). This was most apparent in ADA patients, who showed very high proportions requiring ICU care and severe respiratory support. Across early and late sampled patients, we reasoned that these inconsistencies could be attributed to mechanistic/gene-expression influences that were additional to the endotype signatures in COVID-19 patients (for example, Covid-specific gene expression, COVID-19 specific endotype signatures and/or mixed endotypes).

Comparing clinical data between endotypes indicated that sepsis endotypes might be useful for clinical risk stratification in COVID-19 disease. We next examined global gene expression differences between predicted endotypes to characterize highly dysregulated endotype-specific genes. It is reasonable to hypothesize that these genes might have regulatory functions and could be targeted for endotype-specific therapies. We specifically focused on characterizing the NPS and INF-predicted endotypes of the early sampled group, since detection and treatment would be most impactful in these endotypes and disease stage. DE gene expression analysis was performed comparing each endotype to all others (i.e., in a one vs. rest scheme) to identify global gene expression differences between endotypes. The NPS endotype displayed 2728 (1352 upregulated, 1376 down-regulated) unique DE genes while the INF endotype had 573 (520 upregulated, 53 down-regulated genes) unique DE genes.

**Figure 4:**
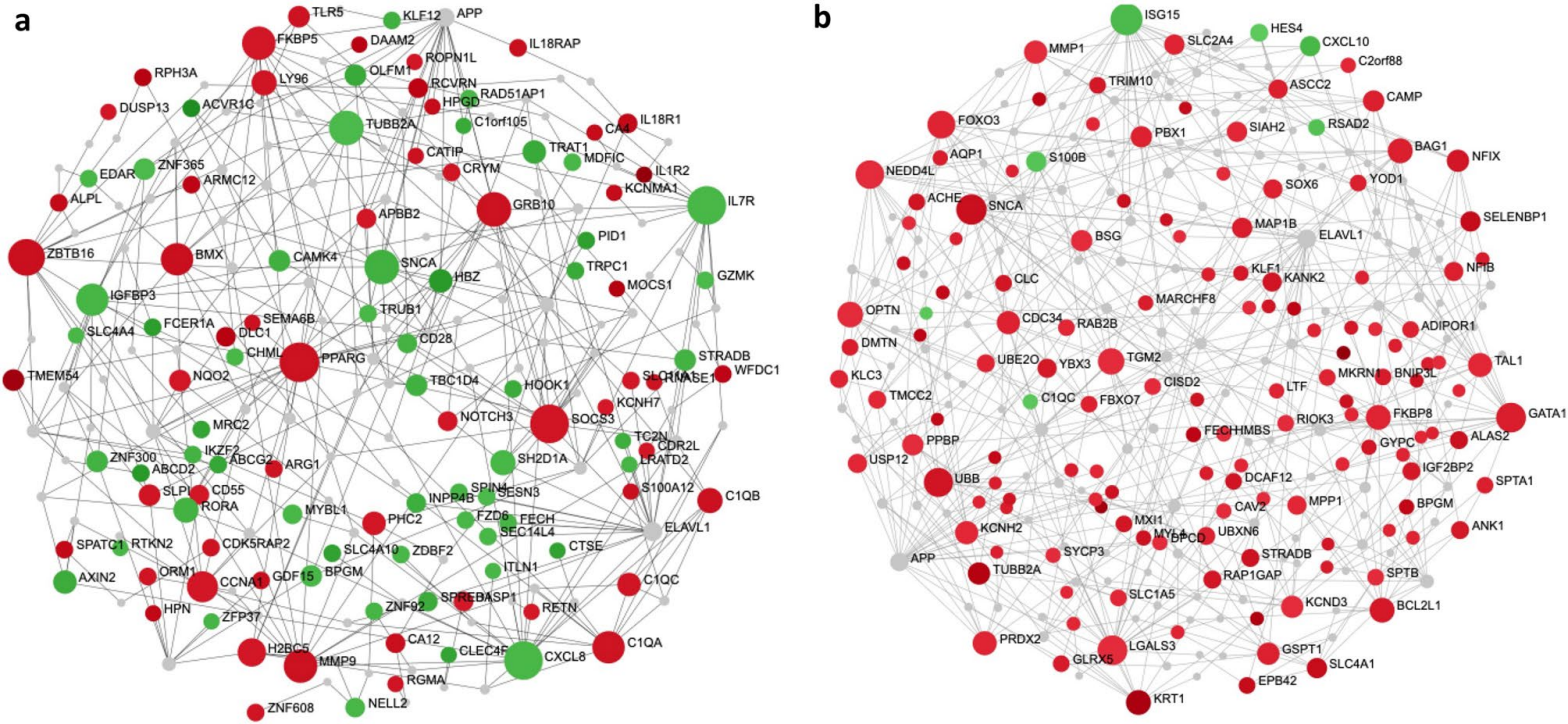
Function-based, minimally connected first order protein:protein interaction networks of the top endotype specific DE genes. (**a**) NPS endotype network. (**b**) INF endotype network. The top 200 endotype-specific DE genes (by absolute value fold changes) were input into NetworkAnalyst^54^. Each DE gene set formed a well-connected minimum connected first-order network, indicating that the genes involved are functionally related and likely collectively regulate or play key roles in one or more related biological mechanisms. Red and green nodes are genes with increased and decreased expression specific to the endotype, respectively, while grey nodes are interconnecting first-order interaction nodes. The size of the nodes indicates their connectivity (hub degree) within the network, i.e., how well interconnected any given node is to other nodes in the Network (NB. hubs are key molecules in signalling since they are highly interconnected; they receive and integrate multiple signals and pass them on to downstream nodes). Lines represent edges that indicate known (experimentally-determined) protein:protein interactions derived from www.innatedb.ca (version 5.4).

These differences were explored in the context of protein:protein interaction (PPI) networks, specifically drawing from the International Molecular Exchange Consortium (ImeX) data, which includes the InnateDB database^33^ (version 5.4) of manually curated immune interactions (Fig. 4). PPI networks reveal known direct-molecular/binding, metabolic or regulatory interactions between individual proteins, and thus represent function-based interactions in cells.

It was apparent that DE genes from the NPS endotype included both up- (red) and down- (green) regulated genes, consistent with the notion that the endotype captures concurrent neutrophil stimulation and immunosuppressive mechanisms (e.g., down-regulated IL-8, IL7R). Of most relevance was the identification of network hubs that indicate highly connected proteins/genes (shown by the size of the nodes/circles) and reflect their importance in the network as key signaling molecules that receive and integrate multiple biological signals. Importantly, these hubs reflect potential endotype-specific drug targets, which, when targeted, could disrupt the network structure and thus the endotype’s pathophysiological basis of severity. Hubs of the NPS endotype network included peroxisome proliferator-activated receptor gamma (PPARG), synuclein alpha (SNCA), suppressor of cytokine signaling 3 (SOCS3), and C-X-C motif chemokine ligand 8/interleukin-8 (CXCL8/IL-8), among others. The INF endotype DE genes were primarily comprised of up-regulated genes. Hubs of the INF network included forkhead box O3 (FOXO3), galectin 3 (LGALS3), transglutaminase 2 (TGM2), ELAV Like RNA Binding Protein 1 (ELAV-1), and interferon-stimulated gene 15 (ISG15). Many of these can be targeted by existing drugs as compiled in Gene-Cards (https://www.genecards.org/) and thus are addressable with repurposed drugs (Supplemental Table 4).

## Discussion

In the current COVID-19 pandemic, severely affected patients have been proposed to suffer from sepsis^34,35^. However, given the enormous heterogeneity of sepsis, it has not been clear if this is a distinct type of sepsis or mechanistically overlapping with all-cause sepsis. Therefore, we analyzed the gene expression profiles of COVID-19 patients to examine dominant gene expression responses. We show that all-cause sepsis signatures of severity, mortality, and mechanisms/endotypes are dysregulated in severe COVID-19 patients, indicating, based on molecular responses, that severe COVID-19 is a form of all-cause sepsis. This is significant because it implies severe COVID-19 can be predicted using the same biomarkers and gene expression signatures useful in all-cause sepsis patients. Furthermore, by identifying their endotypes, Covid-19 patients can be potentially treated with immunomodulatory therapies that correct dysfunctional immune processes specific to a particular endotype, as predicted in Supplemental Table 4. Given several reports indicating that antiviral therapies display no marked efficacy in reducing mortality in COVID-19 patients^36^, this is especially relevant.

We initially identified COVID-19 specific mechanisms that were dysregulated between severity groups (Fig. 1). These results showed that in addition to sepsis-related gene expression (e.g., Neutrophil degranulation, various inflammatory hallmarks), COVID-19 positive patients displayed significant dysregulation of interferon and heme metabolism response that are not as obvious in all-cause sepsis patients. The up-regulation of Type I and II interferon-related pathways in severe COVID-19 has been observed in many previous studies exploring COVID-19 infections^37,38^, and is generally observed in viral infections. COVID-19 patients have also been shown to have excessive and dysfunctional neutrophils^39,40^, although it is worth mentioning that this is a general feature of patients with more severe cases of sepsis^41^.

Heme metabolism was also strongly dysregulated when comparing patients who died vs. survived. In all-cause sepsis, it has been shown that sepsis survivors display a signature of heme biosynthesis, wherein up-regulated heme synthesis prevents further tissue damage from infection^42^. Other studies have shown that severe sepsis patients fail to remove excessive heme leading to tissue damage^28,43^. Some literature exploring heme metabolism in COVID-19 patients indicates that SARS-CoV-2 proteins directly bind hemoglobin inhibiting their metabolism, although this is debated since it has only been posited through in silico experiments^44,45^.

The CR and Organ Dysfunction signatures, which have been shown to predict impending severe sepsis^11,12^, performed well in discriminating between severe, intermediate, and moderate patients in early and late sampled patients with COVID-19. Interestingly, the CR signature, which was originally derived from treating PBMCs twice with lipopolysaccharide (a bacterial agonist) yielding an immune tolerance phenotype, was strongly able to discriminate between severity groups. It is unknown whether signatures of this form of immune tolerance are the same irrespective of bacterial or viral stimuli although both bacterial and viral-like molecules can induce such tolerance^46,47^. Nonetheless, our results clearly indicate that the CR signature is strongly associated with severity due to viral infections and thus reflects an important mechanism (monocyte reprogramming to a non-responsive phenotype) of COVID-19 sepsis. Interestingly, a recent publication showed that the stimulation of human blood cells with SARS-CoV-2 proteins induced hallmarks of CR, including reduced proinflammatory cytokine production and T cell proliferation, and enhancement of phagocytosis and tissue remodeling^48^. Furthermore, there have been other studies which have shown through bioinformatic analysis and mathematical models that related biological responses dysregulated during severe Covid-19 infection overlap with that of all-cause sepsis^38,49,50^. The Mortality signature also captured 30-day mortality quite well, indicating that similar mechanisms arise during severe COVID-19 infection and all-cause sepsis when death is impending.

We also explored endotypes within the COVID-19 gene expression data. Endotypes reflect a more recent paradigm to characterize the sepsis syndrome, whereby each endotype captures distinct mechanisms that an individual patient could express. Accordingly, we predicted endotype status among early and late sampled COVID-19 patients. Predictions generated by GSVA enrichment scores indicated that all-cause sepsis endotypes can, to a great extent, explain the heterogeneity of severe COVID-19 disease. Additional complexity was also observed (Fig. 3), perhaps due to COVID-19-driven gene expression and the somewhat varied time of sampling relative to hospital admission. Thus sepsis and/or COVID-19 signatures may exhibit somewhat different behaviour in the earlier and later stages of the disease, which is consistent with previous studies by our group indicating that gene expression trajectories change over two time points taken seven days apart in severe COVID-19 patients (An et al., manuscript submitted).

Results were most consistent with the expected behaviour of endotypes in the early sampled patients, which is reasonable considering that the endotypes were identified in ER patients at first clinical presentation (within 2 h of admission). Specifically, the predicted NPS endotype patients exhibited the highest mortality and disease severity based on respiratory support requirements. Sweeney et al.^51^ presented an analogous result when applying their three-endotype classifier to COVID-19 patients. That study showed that, based on the expression of 33 biomarker genes (5–17 per endotype), the genes were also expressed in COVID-19 patients, and the defining clinical trends for each endotype were mostly maintained. It is worth noting however that our 5 endotype classifier not only classifies patients into mechanistically and to some extent clinically distinct groups, but each of these endotypes can be accurately classified by expression of a diverse set of gene pairs^12^.

In conclusion, we present compelling evidence that COVID-19 is a type of sepsis based on the dysregulation of specific gene expression signatures, with unique COVID-19 specific immune responses also contributing to patient severity. When exploring the expression of previously-described^12^ all-cause sepsis signatures in a broad spectrum of COVID-19 patients, severely ill patients displayed significant dysregulation of these genes. Furthermore, several COVID-19 gene expression responses have known roles in sepsis pathogenesis, including various inflammatory pathways, neutrophil degranulation, interferon α/β signaling and heme metabolism. This can provide prognostic criteria as well as a precision medicine approach to characterize (and possibly treat) the disease. In conclusion, future opportunities for the clinical management of Covid-19 patients can draw from existing and under-development prognostic tools relevant to all-cause sepsis signatures and therapeutic options that emphasize address a dysregulated host immune response.

## Supplementary Information

Supplementary Information.

## Data Availability

Sequencing data and patient details are available at NCBI GEO (Accession Number GSE221234). Other materials, including study protocol, statistical analysis plan, and code is available to anyone who requests. These requests should be directed to REW Hancock (bob@hancocklab.com).
