# Supplementary material for: Predicting severity in COVID-19 disease using sepsis blood gene expression signatures
Source: Sci Rep. 2023 Jan 23;13:1247. doi: 10.1038/s41598-023-28259-y (PMC9868505; doi:10.1038/s41598-023-28259-y)
Supplement: Supplementary file 1 — Supplementary Information. [file 41598_2023_28259_MOESM1_ESM.docx]

**Predicting severity in COVID-19 disease using sepsis blood gene expression signatures**

Arjun Baghela^1^, Andy An^1^, Peter Zhang^2^, Erica Acton^3^, Jeff Gauthier^4^, Elsa Brunet-Ratnasingham^5,6^, Travis Blimkie^1^, Gabriela Cohen Freue^7^, Daniel Kaufmann^6,8^, Amy H.Y. Lee^3^, Roger C. Levesque^4^, Robert E.W. Hancock^1,2^

^1^Department of Microbiology and Immunology, University of British Columbia (UBC), Vancouver, Canada

^2^ Asep Medical, Vancouver, Canada

^3^ Department of Molecular Biology and Biochemistry, Simon Fraser University, Burnaby, Canada

^4^Institut de biologie intégrative et des systèmes (IBIS), Département de microbiologie-infectiologie et d'immunologie, Université Laval, Québec, Québec, Canada

^5^ Département de Microbiologie, Infectiologie et Immunologie, Université de Montréal, Montreal, Canada

^6^Centre de Recherche du CHUM, Montreal, QC, H2X 0A9 Canada

^7^ Department of Statistics, UBC, Vancouver, Canada

^8^ Département de Médecine, Université de Montréal, Montreal, Canada

**SUPPLEMENTAL MATERIAL**

**Supplemental Table 1 – Gene-set differential expression of the Full Cellular Reprogramming (CR; 31 genes), Organ Dysfunction (52 genes), and Mortality (38 genes) signatures in COVID-19 patient cohorts.** The gene sets were assessed for significant dysregulation between patients using ROAST. Statistically significant gene-set DE is indicated by bold typeface (FDR adjusted P ≤ 0.05). Bold text shows statistically significant data while red indicates differences from the reduced signatures. The reduced equivalents described in Table 1 in the main text were identified by further filtering the genes input to lasso using a more stringent fold change cut off (top 25% highest fold changes). These were identified to facilitate more rapid and accessible translation to clinics.

| **Cohort** | **Comparison** | **Statistical significance (p-value)** | | |
| --- | --- | --- | --- | --- |
|  |  | **CR** | **Organ Dysfunction** | **Mortality** |
| **All** | Severe (54) vs. Moderate (42) | **< 0.0001** | **< 0.0001** | **< 0.0001** |
|  | Severe (54) vs. Intermediate (28) | **0.0039** | **0.00020** | **0.0050** |
|  | Intermediate (28) vs. Moderate (42) | **0.00070** | **< 0.0001** | **0.0011** |
|  | Died (20) vs. Survived (104) | **< 0.0001** | **< 0.0001** | **< 0.0001** |
| **Early Sampled (0-5 days)** | Severe (28) vs. Moderate (29) | **< 0.0001** | **< 0.0001** | **< 0.0001** |
|  | Severe (28) vs. Intermediate (19) | **0.028** | **0.010** | **0.019** |
|  | Intermediate (19) vs. Moderate (29) | **0.0020** | **< 0.0001** | **0.0077** |
|  | Died (11) vs. Survived (65) | **0.0013** | **< 0.0001** | **< 0.0001** |
| **Late Sampled (6-20 days)** | Severe (26) vs. Moderate (13) | **0.00050** | **< 0.0001** | **0.00030** |
|  | Severe (26) vs. Intermediate (9) | 0.11 | **0.0035** | 0.23 |
|  | Intermediate (9) vs. Moderate (13) | 0.11 | **0.040** | **0.071** |
|  | Died (9) vs. Survived (39) | **0.0012** | **0.0079** | **0.0011** |

**Supplemental Table 2 – Clinical differences between predicted endotypes in the early sampled group.** The mean value ± standard error is presented for numerical variables with the total available observations/patient numbers recorded in brackets. Categorical variables are presented as % positive (relative to total available observations). Significance was assessed using a Kruskal-Wallis and Chi Square Test for numeric and categorical parameters, respectively.

| **Parameter** | **NPS (17/76)** | **INF (16/76)** | **IHD (12/76)** | **IFN (14/76)** | **ADA (17/76)** | **P Value** |
| --- | --- | --- | --- | --- | --- | --- |
| Respiratory Support Group - Moderate | 5.9% (1/17) | 25% (4/16) | 91.7% (11/12) | 50.0% (7/14) | 35.3% (6/17) | 0.000075 |
| Receiving ICU care | 76.5% (13/17) | 62.5% (10/16) | 8.3% (1/12) | 14.3% (2/14) | 29.4% (5/17) | 0.00018 |
| Respiratory Support Group - Severe | 76.5% (13/17) | 37.5% (6/16) | 8.3% (1/12) | 21.4% (3/14) | 29.4% (5/17) | 0.0015 |
| Glasgow Coma Score | 13.3 ± 1.71 (7) | 8 ± 2.86 (5) | 15 ± 0 (5) | 15 ± 0 (5) | 15 ± 0 (9) | 0.0174 |
| P/F Ratio | 213.6 ± 19.8 (7) | 175.2 ± 29.9 (5) | 382.6 ± 21.4 (5) | 274.4 ± 58.3 (5) | 254.8 ± 31.26 (9) | 0.022 |
| qSOFA | 0.6 ± 0.3 (7) | 1.4 ± 0.4 (5) | 0 ± 0 (5) | 0.2 ± 0.2 (5) | 0.4 ± 0.18 (8) | 0.0454 |
| Sampling to Hospital Admission (days) | 2.9 ± 0.31 (17) | 3.5 ± 0.32 (16) | 3.4 ± 0.34 (12) | 2.8 ± 0.3 (14) | 4.0 ± 0.26 (17) | 0.053 |
| Receiving Antibiotics | 94.1% (16/17) | 75% (12/16) | 45.5% (5/11) | 71.4% (10/14) | 76.5% (13/17) | 0.078 |
| Chronic Lung Insufficiency | 0% | 31.2% (5/16) | 8.3% (1/12) | 21.4% (3/14) | 11.8% (2/17) | 0.11 |
| Diastolic Blood Pressure | 63.4 ± 5.45 (7) | 61.4 ± 7.41 (5) | 81.2 ± 5.91 (5) | 73 ± 3.97 (5) | 75.1 ± 3.71 (9) | 0.1229 |
| Sampling to Symptom Onset (days) | 8.7 ± 0.84 (15) | 10.7 ± 1.36 (16) | 11 ± 1.3 (12) | 8.6 ± 1.02 (14) | 11.4 ± 0.88 (17) | 0.1346 |
| Non-COVID Active Infection | 58.8% (10/17) | 25% (4/16) | 25% (3/12) | 21.4% (3/14) | 23.5% (4/17) | 0.1 |
| Type 2 Diabetes | 52.9% (9/17) | 43.8% (7/16) | 8.3% (1/12) | 35.7% (5/14) | 29.4% (5/17) | 0.15 |
| Heart Rate (beats/min) | 80 ± 5.09 (7) | 71.8 ± 3.44 (5) | 89.2 ± 6.71 (5) | 76.4 ± 5.57 (5) | 77.8 ± 3.5 (9) | 0.1963 |
| Time in Hospital (days) | 25.1 ± 6.12 (17) | 17.2 ± 2.93 (16) | 15 ± 7.5 (12) | 19.2 ± 5.03 (14) | 15.6 ± 2.95 (17) | 0.2209 |
| PaO2 mm Hg | 79.4 ± 5.18 (7) | 70.8 ± 5.34 (5) | 80.4 ± 4.39 (5) | 68.4 ± 6.5 (5) | 74 ± 3.11 (9) | 0.2279 |
| SpO2 mm Hg | 95 ± 0.72 (7) | 93.2 ± 1.11 (5) | 95.4 ± 0.68 (5) | 92 ± 1.82 (5) | 94.1 ± 0.7 (9) | 0.2279 |
| Chronic Heart Insufficiency | 0% | 0% | 16.7% (2/12) | 35.7% (5/14) | 0% | 0.27 |
| SOFA | 8.7 ± 0.98 (12) | 9 ± 1.27 (7) | 11 ± 0.0 (1) | 10 ± 3.51 (3) | 5.3 ± 1.71 (7) | 0.2835 |
| Respiratory Support Group - Intermediate | 17.6% (3/17) | 37.5% (6/16) | 0% | 28.6% (4/14) | 35.3% (6/17) | 0.30 |
| AVPU - Unresponsive | 14.3% (1/7) | 40% (2/5) | 0% | 0% | 0% | 0.31 |
| Dyslipidemia | 58.8% (10/17) | 33.3% (5/15) | 25% (3/12) | 50% (7/14) | 35.3% (6/17) | 0.34 |
| Systolic Blood Pressure (mmHG) | 138 ± 5.46 (7) | 127.6 ± 7.05 (5) | 129.6 ± 9.69 (5) | 116.2 ± 8.14 (5) | 131.4 ± 8.35 (9) | 0.3451 |
| Mortality | 29.4% (5/17) | 6.2% (1/16) | 8.3% (1/12) | 14.3% (2/14) | 11.8% (2/17) | 0.38 |
| Sex (F) | 47.1% (8/17) | 50% (8/16) | 66.7% (8/12) | 28.6% (4/14) | 41.2% (7/17) | 0.43 |
| Previous/Resolved Cancer | 6.2% (1/16) | 18.8% (3/16) | 8.3% (1/12) | 0% | 5.9% (1/17) | 0.44 |
| Admission to Symptom Onset (days) | 6 ± 0.76 (15) | 7.2 ± 1.36 (16) | 7.6 ± 1.21 (12) | 5.8 ± 1.11 (14) | 7.4 ± 0.8 (17) | 0.4935 |
| Temperature (Celsius) | 37.3 ± 0.31 (7) | 36.7 ± 0.31 (5) | 36.6 ± 0.18 (5) | 37.2 ± 0.31 (5) | 37 ± 0.24 (9) | 0.515 |
| Age (years) | 63.6 ± 3.74 (17) | 64.1 ± 3.54 (16) | 56.2 ± 4.39 (12) | 65.4 ± 3.72 (14) | 61.3 ± 4.23 (17) | 0.6297 |
| Active Cancer | 12.5% (2/16) | 6.2% (1/16) | 8.3% (1/12) | 14.3% (2/14) | 0% | 0.63 |
| Chronic Renal Insufficiency | 23.5% (4/17) | 25% (4/16) | 16.7% (2/12) | 28.6% (4/14) | 17.6% (3/17) | 0.95 |

**Supplemental Table 3 – Clinical differences between predicted endotypes in the late sampled group.** The mean value ± standard error is presented for numerical variables with the total available observations/patient numbers recorded in brackets. Categorical variables are presented as % positive (relative to total available observations). Significance was assessed using a Kruskal-Wallis and Chi Square Test for numeric and categorical parameters, respectively.

| **Parameter** | **NPS (10/48)** | **INF (13/48)** | **IHD (10/48)** | **IFN (9/48)** | **ADA (6/48)** | **P Value** |
| --- | --- | --- | --- | --- | --- | --- |
| Mortality | 60% (6/10) | 0% | 0% | 11.1% (1/9) | 33.3% (2/6) | 0.0010 |
| Respiratory Support Group - Severe | 90% (9/10) | 61.5% (8/13) | 20.0% (2/10) | 33.3% (3/9) | 66.7% (4/6) | 0.012 |
| Age (years) | 65.9 ± 2.77 (10) | 58.2 ± 4.26 (13) | 51.8 ± 4.64 (10) | 68.7 ± 5.01 (9) | 51 ± 4.72 (6) | 0.0323 |
| Chronic Renal Insufficiency | 30% (3/10) | 7.7% (1/13) | 0% | 44.4% (4/9) | 0% | 0.045 |
| Receiving ICU Care | 90% (9/10) | 61.5% (8/13) | 30% (3/10) | 44.4% (4/9) | 83.3% (5/6) | 0.049 |
| Glasgow Coma Score | 5.4 ± 2.4 (5) | 12 ± 3 (4) | 15 ± 0 (3) | 15 ± 0 (3) | 13.5 ± 1.5 (4) | 0.0645 |
| qSOFA | 1.8 ± 0.49 (5) | 0.8 ± 0.48 (4) | 0 ± 0 (3) | 0 ± 0 (3) | 0.5 ± 0.29 (4) | 0.0706 |
| AVPU - Unresponsive | 80% (4/5) | 25% (1/4) | 0% | 0% | 0% | 0.098 |
| Type 2 Diabetes | 60% (6/10) | 15.4% (2/13) | 40% (4/10) | 66.7% (6/9) | 50% (3/6) | 0.11 |
| SOFA Score | 10.4 ± 1.09 (7) | 8 ± 1.15 (7) | 3.7 ± 2.67 (3) | 8 ± 3.06 (3) | 6 ± 2.04 (4) | 0.1161 |
| P/F Ratio | 192.4 ± 41.46 (5) | 209.5 ± 39.22 (4) | 339.3 ± 27.02 (3) | 361 ± 37.72 (3) | 218.5 ± 67.61 (4) | 0.1165 |
| PaO2 mm Hg | 86.2 ± 5.45 (5) | 67 ± 3.14 (4) | 74.7 ± 5.24 (3) | 77 ± 7.09 (3) | 73 ± 4.06 (4) | 0.1202 |
| SpO2 mm Hg | 96.2 ± 0.58 (5) | 92.5 ± 0.96 (4) | 94.3 ± 1.2 (3) | 94.7 ± 1.2 (3) | 94 ± 0.91 (4) | 0.1202 |
| Sampling to Hospital Admission (days) | 10.2 ± 1.13 (10) | 11.2 ± 1.31 (13) | 8.3 ± 1.04 (10) | 8 ± 1.04 (9) | 7.2 ± 0.65 (6) | 0.1208 |
| Admission to Discharge (days) | 36 ± 5.12 (10) | 26 ± 5.97 (13) | 24.2 ± 10.58 (10) | 40.3 ± 11.17 (9) | 26.2 ± 7.65 (6) | 0.1601 |
| Sampling to Symptom Onset (days) | 16.7 ± 1.28 (10) | 17.3 ± 1.43 (11) | 14.8 ± 1.61 (9) | 14.1 ± 2.68 (9) | 12.4 ± 0.68 (5) | 0.161 |
| Chronic Heart Insufficiency | 30% (3/10) | 0% | 10% (1/10) | 22.2% (2/9) | 0% | 0.17 |
| Diastolic Blood Pressure | 56 ± 5.82 (5) | 71.2 ± 7.05 (4) | 77.7 ± 2.33 (3) | 74.7 ± 2.33 (3) | 68.8 ± 6.02 (4) | 0.1746 |
| Systolic Blood Pressure | 122 ± 8.04 (5) | 144.5 ± 5.07 (4) | 128.3 ± 2.4 (3) | 135.3 ± 15.07 (3) | 120.5 ± 6.25 (4) | 0.1799 |
| Previous/Resolved Cancer | 0% | 7.7% (1/13) | 0% | 22.2% (2/9) | 0% | 0.18 |
| Respiratory Support Group - Moderate | 0% | 23.1% (3/13) | 60.0% (6/10) | 33.3% (3/9) | 16.7% (1/6) | 0.21 |
| Non-COVID Active Infection | 50% (5/10) | 53.8% (7/13) | 20% (2/10) | 62.5% (5/8) | 16.7% (1/6) | 0.22 |
| Chronic Lung Insufficiency | 0% | 7.7% (1/13) | 0% | 0% | 16.7% (1/6) | 0.41 |
| Sex (F) | 20% (2/10) | 38.5% (5/13) | 10% (1/10) | 44.4% (4/9) | 16.7% (1/6) | 0.42 |
| Temperature (Celsius) | 36.5 ± 0.23 (5) | 37 ± 0.14 (4) | 36.4 ± 0.17 (3) | 36.7 ± 0.34 (3) | 36.4 ± 0.43 (4) | 0.4795 |
| Receiving Antibiotics | 100% (10/10) | 84.6% (11/13) | 77.8% (7/9) | 85.7% (6/7) | 100% (6/6) | 0.55 |
| Dyslipidemia | 50% (5/10) | 23.1% (3/13) | 30% (3/10) | 44.4% (4/9) | 50% (3/6) | 0.62 |
| Respiratory Support Group - Intermediate | 10% (1/10) | 15.4% (2/13) | 20.0% (2/10) | 33.3% (3/9) | 16.7% (1/6) | 0.76 |
| Admission to Symptom Onset (days) | 6.5 ± 1.3 (10) | 6 ± 0.99 (11) | 7.4 ± 1.32 (9) | 6.1 ± 1.72 (9) | 5 ± 0.95 (5) | 0.8265 |
| Heart Rate (beats/min) | 74.8 ± 6.79 (5) | 74.5 ± 5.56 (4) | 81 ± 3.51 (3) | 80 ± 8.96 (3) | 83 ± 7.93 (4) | 0.9151 |

**Supplemental Table 4 – Approved drug candidates for protein products of endotype-specific DE genes.** These targets were the most highly-connected nodes (i.e. hubs) in the endotype specific DE gene PPI networks presented in **Figure 4**, reflecting their importance as key molecules in signalling. Hub degree (i.e. the Degree of Connectivity) indicates how many other nodes the hub is connected with through PPIs. Drug targeting data was obtained from Gene-Cards (<https://www.genecards.org/)>.

| **Network Gene/ Target** | **Fold Change** | **Hub Degree** | **Drug Candidates for Target** |
| --- | --- | --- | --- |
| **NPS Network** | | | |
| PPARG | 2.73 | 17 | Rosiglitazone, Pioglitazone, Troglitazone, Bezafibrate, Indomethacin, Mesalazine, Bisphenol A diglycidyl ether, Fenofibric acid, Bexarotene, Balsalazide, Sulfasalazine, Repaglinide, Telmisartan, Curcumin, Ibuprofen, Ciprofibrate, Fenofibrate, Gemfibrozil, Acetylsalicylic acid, Olanzapine, Diclofenac, Zafirlukast, Celecoxib, Levothyroxine, Linoleic acid, Glipizide, Nateglinide, Icosapent, Amiodarone, Cannabidiol, Clofazimine, Dexibuprofen, Doconexent, Fenoprofen, Flufenamic acid, Oleic Acid, Triclosan, Valproic acid, alpha-Linolenic acid, Fish oil, Omega-3 fatty acids, Estradiol, Gentian violet cation, Glyburide, Methotrexate, Methylene blue, Mycophenolic acid, Phenolphthalein, Propylparaben, Raloxifene, Spironolactone, Treprostinil, Zoledronic acid, Atorvastatin, Palmitic Acid, Stearic acid, Acarbose, Amprenavir, Beta-D-Glucose, Cholesterol, Clofibrate, Dexamethasone, Diethylstilbestrol, Epitestosterone, Eprosartan, Gamolenic acid, Glimepiride, Glycerin, Indinavir, Irbesartan, Losartan, Metformin, Midazolam, Nimesulide, Orlistat, Phenobarbital, Pravastatin, Ritonavir, Rofecoxib, Saquinavir, Simvastatin, Sirolimus, Stavudine, Sulindac, Tacrolimus, Tocopherol, Valsartan, Cholecalciferol, Isotretinoin, L-Glutamine, Niacin |
| CXCL8 | -2.45 | 17 | Bevacizumab, Sunitinib, Clarithromycin, Pentoxifylline, Troglitazone, Simvastatin, Acetaminophen, Acetylsalicylic acid, Alprazolam, Ceftriaxone, Cetuximab, Cidofovir, Colchicine, Dacarbazine, Danazol, Dipyridamole, Esomeprazole, Fentanyl, Foscarnet, Hydroquinone, Ibuprofen, Lansoprazole, Leflunomide, Methimazole, Midazolam, Naproxen, Paclitaxel, Pamidronic acid, Ribavirin, Talc, Terfenadine, Verapamil, Isotretinoin, Vitamin A, Salbutamol, Aprotinin, Arformoterol, Azithromycin, Budesonide, Calcipotriol, Cetirizine, Curcumin, Cyclosporine, Dactinomycin, Desloratadine, Dexamethasone, Echinacea, Emedastine, Erythromycin, Etanercept, Fluticasone propionate, Heparin, bovine, Histamine, Hyaluronic acid, Hydrocortisone, Imiquimod, Levocetirizine, Loratadine, Methacholine, Mifepristone, Montelukast, Moxifloxacin, Nitric Oxide, Nonoxynol-9, Olopatadine, Oxygen, Parthenolide, Polymyxin B, Rebamipide, Roxithromycin, Salmeterol, Sulfapyridine, Sulfasalazine, Cysteine |
| IL7R | -2.50 | 16 | Bovine heparin, D-Tyrosine, Herbimycin A, Ionomycin |
| SOCS3 | 2.53 | 16 | Beta-D-Glucose, Cocaine, Diethylstilbestrol, Epitestosterone, Nitric Oxide, Ribavirin, Rosiglitazone, D-Lysine, D-Threonine, D-Tyrosine |
| ZBTB16 | 2.86 | 14 | Zinc cation, Epitestosterone, Progesterone, D-Tyrosine, Isotretinoin |
| **INF** | | | |
| ISG15 | -3.77 | 22 | Irinotecan, Progesterone, Cysteine, D-Lysine, Isotretinoin |
| LGALS3 | 2.62 | 19 | Lactose, Acetylsalicylic acid, Cisplatin, Diethylstilbestrol, Etoposide, Formaldehyde, Galactose, Heparin, bovine, Nitric Oxide, Oxygen, Pectin, Progesterone, Sucrose, Cholecalciferol, Cysteine, D-Threonine, D-Tyrosine, Isotretinoin, L-Glutamine |
| SNCA | 4.56 | 19 | Copper, Gentian violet cation, Ketoconazole, Mycophenolic acid, Acetylcholine, Apomorphine, Atropine, Beta-D-Glucose, Carbamoylcholine, Cholesterol, Cocaine, Dextroamphetamine, Doconexent, Dopamine, Doxycycline, Epitestosterone, Ethanol, Heparin, bovine, Iron, Levodopa, Magnesium, Metamfetamine, Nitric Oxide, Norepinephrine, Oxygen, Polyethylene glycol 3500, Rifampicin, Selegiline, Silver cation, Sirolimus, Tacrolimus, Choline, Cysteine, D-Lysine, D-Tyrosine, Icosapent, Lecithin, L-Glutamine, Melatonin, Racemethionine, Valine |
| GATA1 | 3.45 | 18 | Cytarabine, Daunorubicin, Aminolevulinic acid, Azathioprine, Chloramphenicol, Dactinomycin, Diethylstilbestrol, Doxorubicin, Hydroxyurea, Iron, Progesterone, Troglitazone, Zinc cation, Cholecalciferol, Isotretinoin |
| NEDD4L | 2.49 | 16 | Citalopram, Hydrochlorothiazide, Phosphoric acid, Pyrophosphoric acid, Adenosine phosphate, Cysteine |


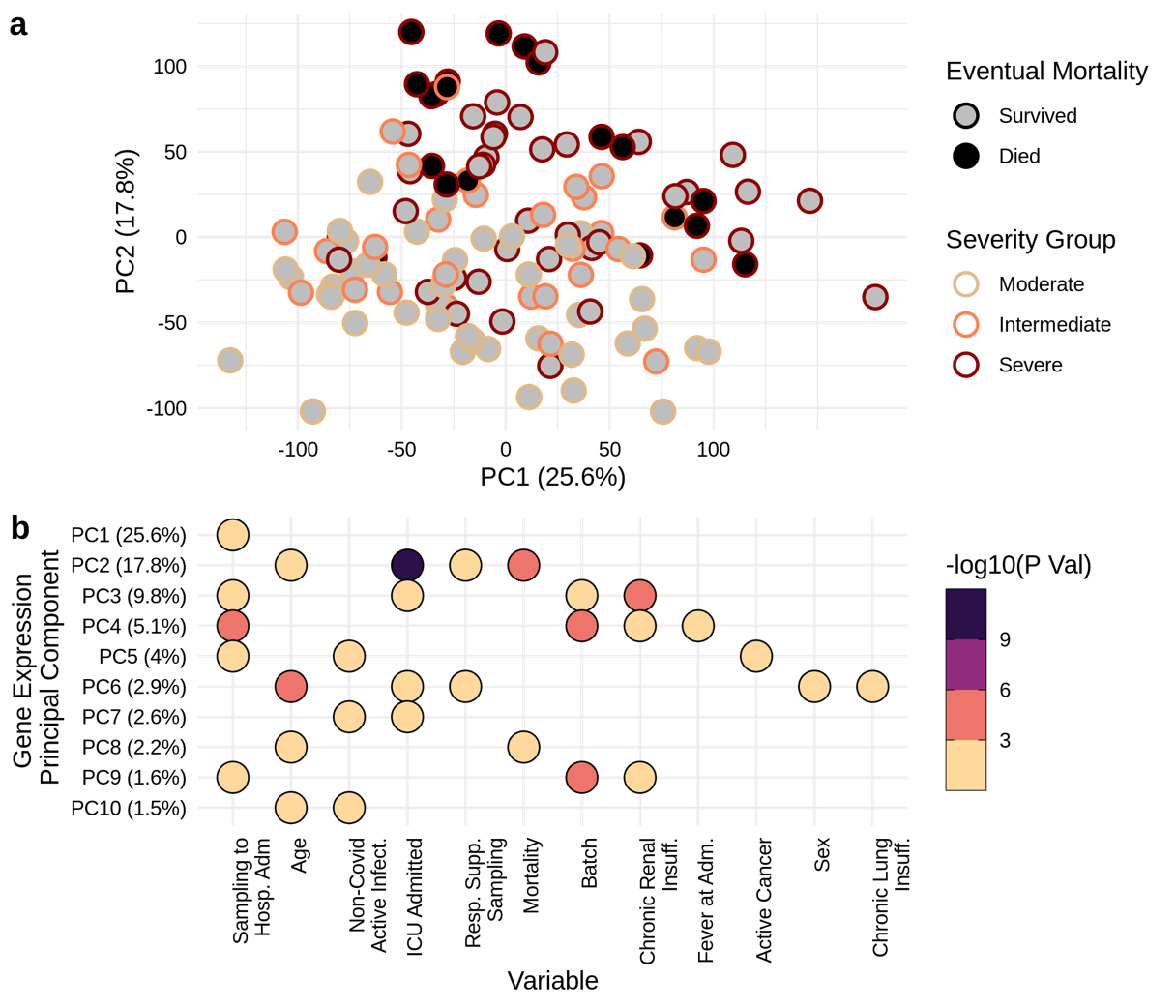


**Supplemental Figure 1 – Principal Component Analysis (PCA) performed on the gene expression profiles of COVID-19 patients.** (a) Two-dimensional view (i.e., PC1 and PC2) of patient gene expression profiles. Severity group (shape outline color) and eventual mortality (shape fill color) is indicated. (b) Association of patient severity and other parameters (e.g., age, antibiotic use, sex, severity, comorbidities, etc.) with the gene expression PCs (PC1-10). Each PC was used in a multiple linear regression as a response variable, while patient outcomes and parameters were used as covariates. For each covariate, the coefficient p-value was extracted to represent its association with each PC. Associations with p-values < 0.05 are presented. Time of sampling relative to hospital admission, age, maximum respiratory support at sampling, ICU admission, and eventual mortality showed significant association to PC1 and PC2, which capture the most variables genes within the gene expression data.


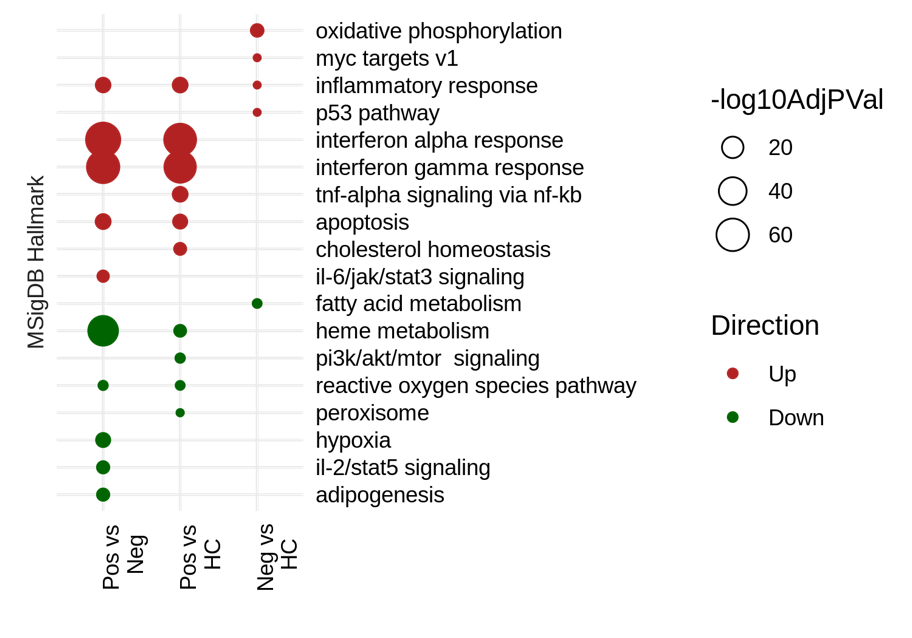


**Supplemental Figure 2 – Functional characterization of gene expression differences between COVID-19 positive, negative, and healthy controls.** Using gene expression data previously published [12] (Baghela et al. 2022; GEO accession GSE185263), the COVID-19 positive vs. negative, positive vs. healthy control, negative vs. healthy control comparisons yielded 2,380 (1,086 up, 1,294 down), 1,327 (568 up, 759 down), and 246 (114 up, 132 down) DE genes, respectively. When comparing COVID-19 positive patients to healthy controls, these hallmarks were mostly maintained. These same hallmarks were not observed when comparing Covid-negative patients to healthy controls. These results showed that in addition to sepsis-related gene expression, COVID-19 positive patients had unique dysregulated mechanisms, most notably interferon and heme metabolism responses.


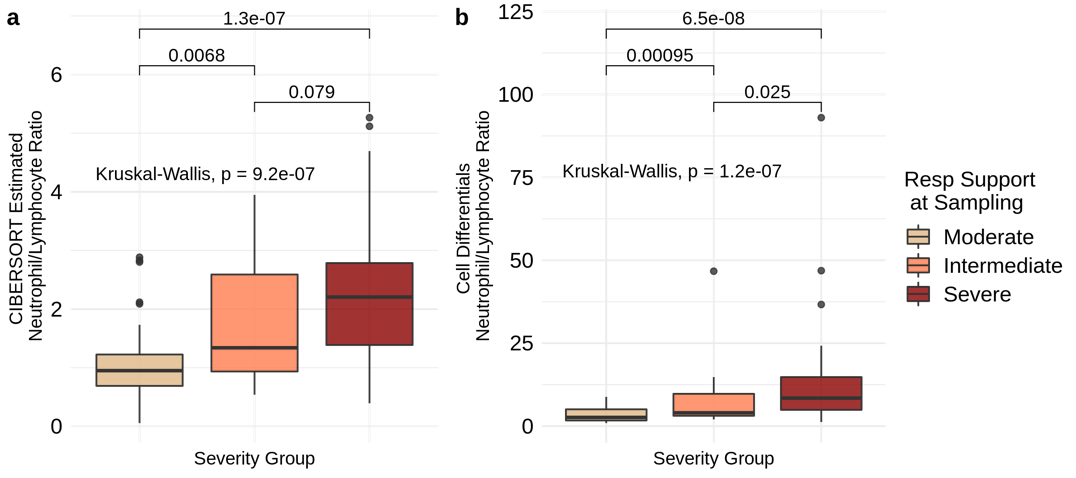


**Supplemental Figure 3. Neutrophil to lymphocyte ratios of severity groups estimated using CIBERSORT and measured cell differentials collected in hospital.** (a) The deconvolution tool CIBERSORT was used to estimate the cell proportions for 22 cell types directly from gene expression profiles. Severe patients displayed the highest ratios and were significantly different from moderate patients by pairwise Wilcoxon test, but not from intermediate patients that were also significantly different from moderate patients. (b) The neutrophil to lymphocyte ratio was also calculated using cell differential values measured at sampling. Cell differentials were available for 108 patients. Ratios were skewed more across severity groups when calculated from cell differentials, but significance testing yielded similar results. Importantly, both CIBERSORT estimated and cell differential ratios were significantly correlated (r = 0.59; p-value = 2.3 x10^-11^).

**Supplemental Figure 4 – Differential expression and functional analysis of COVID-19 patients with and without existing chronic organ insufficiencies (comorbidities) at sampling.** (a) Upset plot presenting the overlap of genes dysregulated between comparisons (i.e., heart insufficiency vs. no heart insufficiency, etc.). The DE genes displayed ≥ ±1.5-fold change and adjusted p ≤ 0.01. Age, sex, antibiotic usage, time of sampling relative to hospital admission, respiratory support severity group, as well as chronic insufficiencies not being directly compared were included in the underlying DESeq2 model to correct for their contribution to gene expression. The chronic renal insufficiency vs. no renal insufficiency, chronic lung insufficiency vs. no lung insufficiency, and chronic heart insufficiency vs. no heart insufficiency yielded 341 (274 up, 67 down), 537 (408 up, 129 down), and 76 (17 up, 59 down) DE genes, respectively. It is important to note that due to low sample sizes in patients with comorbidities, these results require further validation for accuracy and reproducibility. (b) Functional characterization of DE genes using an over-representation analysis of MSigDB sets (adjusted p-value≤0.05).


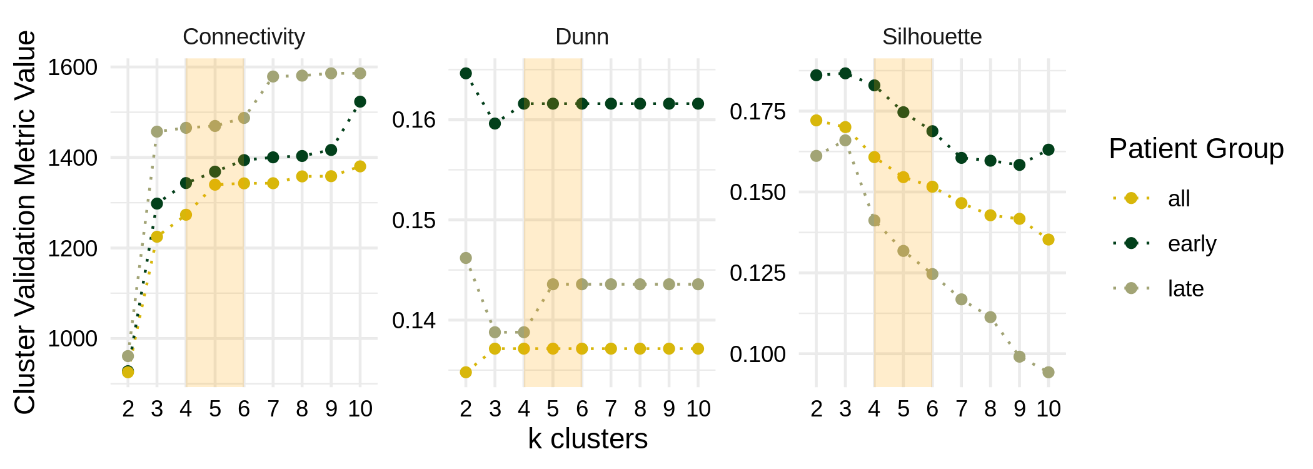


**Supplemental Figure 5 – Applying cluster validity metrics to the gene expression profiles to determine the optimal number of clusters.** Typically, when selecting the appropriate number of clusters using these validity metrics, the *k* at which an elbow arises is considered the optimal number of clusters. This is a heuristic approach which indicates the *k* value whereby further increases of *k* do not explain significant increases in within/between cluster variability.
